# Direct Fabrication of CsPb_x_Mn_1−x_(Br,Cl)_3_ Thin Film by a Facile Solution Spraying Approach

**DOI:** 10.3390/nano11123242

**Published:** 2021-11-29

**Authors:** Yu Sun, Jin Chen, Fengchao Wang, Yi Yin, Yan Jin, Jun Wang, Xiaogai Peng, Ruiyi Han, Canyun Zhang, Jinfang Kong, Jing Yang

**Affiliations:** 1 Shanghai Institute of Technology, College of Sciences, 100 Haiquan Road, Shanghai 201418, China; yusunwei@126.com (Y.S.); particularlymin@outlook.com (Y.Y.); jinyan@sit.edu.cn (Y.J.); wangj@sit.edu.cn (J.W.); ruiyihan39@163.com (R.H.); zhang_canyun@sit.edu.cn (C.Z.); jfkong@sit.edu.cn (J.K.); yangjingxqq@sit.edu.cn (J.Y.); 2 State Key Laboratory of Transducer Technology, Shanghai Institute of Microsystem and Information Technology, Chinese Academy of Sciences, Shanghai 200050, China; 3 State Key Laboratory of Precision Spectroscopy, School of Physics and Electronic Science, East China Normal University, Shanghai 200062, China; pengxiaogai@163.com

**Keywords:** MnCl_2_ doping, CsPb_x_Mn_1−x_(Br,Cl)_3_, thin films, solution spraying

## Abstract

Nowadays, Mn-doping is considered as a promising dissolution for the heavy usage of toxic lead in CsPbX_3_ perovskite material. Interestingly, Mn-doping also introduces an additional photoluminescence band, which is favorable to enrich the emission gamut of this cesium lead halide. Here, a solution spraying strategy was employed for the direct preparation of CsPb_x_Mn_1−x_(Br,Cl)_3_ film through MnCl_2_ doping in host CsPbBr_3_ material. The possible fabrication mechanism of the provided approach and the dependences of material properties on Mn-doping were investigated in detail. As the results shown, Pb was partially substituted by Mn as expected. With the ratio of PbBr_2_:MnCl_2_ increasing from 3:0 to 1:1, the obtained film separately featured green, cyan, orange-red and pink-red emission, which was caused by the energy transferring process. Moreover, the combining energy of Cs, Pb, and Mn gradually red-shifted resulted from the formation of Cs-Cl, Pb-Cl and Mn-Br coordination bonding as MnCl_2_ doping increased. In addition, the weight of short decay lifetime of prepared samples increased with the doping rising, which indicated a better exciton emission and less defect-related transition. The aiming of current work is to provide a new possibility for the facile preparation of Mn-doping CsPbX_3_ film material.

## 1. Introduction

All-inorganic CsPbX_3_ (X=Cl, Br, or I) perovskite material has become a promising candidate for light emitting diodes (LEDs) [1], solar cells [2], lasers [3,4] and so forth [5,6] due to their superior optical properties [7], such as high color purity [8], high photo-luminescence quantum yield (PLQY) [5], excellent carrier characteristics [9] and tunable band gap [10]. Unfortunately, the toxicity of lead is a great challenge for the development of lead halide perovskite [11,12]. How to reduce or replace lead by less-toxic or non-toxic metals is one of the key issues for its commercial application. Currently, great efforts have been made to partially or completely replace Pb by doping divalent cations (such as Sn^2+^, Ge^2+^, Zn^2+^) as the core elements of CsPbX_3_ material [13]. However, the susceptible oxidation of Sn and Ge from bivalent state to tetravalent state exposed under ambient environment is risky for the material stability. Regarding Zn^2+^, the doping concentration in the host material is limited, which is unfavorable to further eliminate the toxic Pb use [14]. 

Interestingly, Mn-doped CsPbX_3_ material has new pleasant chemical and physical properties. It has become a promising candidate for lead-less perovskite material [15]. As suggested by previous reports [16], Mn^2+^ doping can improve the material stability, which derives from Mn-X bond having higher binding energy than Pb-X bond. The most interesting result after partial substitutions of Mn for Pb element is that there is an additional photoluminescence (PL) band in the orange region compared with the pure CsPbX_3_ material [17,18]. Commonly, Mn-doped CsPbX_3_ can be fabricated by halide exchange driven cation exchange strategy [19,20]. Sheldon and co-workers reported the first attempt at Mn-doping based on host material of CsPbCl_3_ by hot injection method [17]. Later, Zhu et al. found that Mn also can doped into CsPbBr_3_ structure via MnCl_2_ to form mixed halide perovskites in 2017 [21]. These mixed halide perovskites can support a much higher Mn doping ratio while retaining the original perovskite emission [15,16,22]. Furthermore, Cl-to-Br anion exchange was adopted to modify the compositions of Mn-doped perovskite material, leading to multi-color luminescence [22]. It is known that the methods of hot injection [23], thermal solvent [24], and anti-solvent [20] are prevalent for the fabrication of Mn-doping CsPbX_3_ material. However, these approaches are limited by the shortcomings of low yields, complex procedures, and toxic chemicals. In addition, the obtained product prepared by the above methods is in a powder-state rather than in film-state. It is well known that the solution spraying method is widely used for the multi-element material fabrication, such as ZnS [25], Cu_2_SnS_3_ [26], Cu_2_ZnSnS_4_ [27], etc., due to its prominent merits of low cost, simple operation, low time-consuming and scalable production. Yang et al. reported their work about the fabrication of CsPbBr_3_ thin film through a spraying method combined with raw CsPbBr_3_ nanocrystals (NCs) [28]. However, the direct deposition for this perovskite film by spraying strategy is rare to date, especially in CsPb_x_Mn_1−x_(Br,Cl)_3_ film material.

Herein, the solution spraying strategy was employed for the direct preparation of CsPb_x_Mn_1−x_(Br,Cl)_3_ film through MnCl_2_ doping in host CsPbBr_3_ material for the first time. The soft polyethylene terephthalate (PET) material was used as the substrate. It is well known that the flexible substrate features distinguished plasticity and tailorability, which is favorable to widen the application of prepared CsPb_x_Mn_1−x_(Br,Cl)_3_ material such as curved displaying or wearable devices [29]. In the current work, the influences of MnCl_2_ doping on the material properties including photoluminescence, structure, morphology, chemical composition, carrier decay lifetime, etc., were investigated in detail. Moreover, the fabrication mechanism of the proposed spraying approach was also analyzed. This work aims to provide a new possibility for the facile preparation of Mn-doping CsPbX_3_ film material.

## 2. Experimental Details

### 2.1. Preparation of CsPbBr_3_ and CsPb_x_Mn_1−x_(Br,Cl)_3_ Thin Films

Typically, the precursors were prepared by 0.2 mmol CsBr (cesium bromide, 99%, AR), 0.2 mmol PbBr_2_ (lead bromide, 99%, AR), 40 ul EAC (ethyl acetate, 90%, AR), 40 ul OLA (oleylamine, 70%, AR) and 10 mL DMSO (Dimethyl sulfoxide, 90%) were successively added into the beaker with stirring until reactants were dissolved sufficiently. Then, the transparent solution was poured into spraying devices, and sprayed onto flexible PET substrate at 110 ℃ within 60 s to obtain the expected sample. Concerning to the fabrication of CsPb_x_Mn_1−x_(Br,Cl)_3_ films, MnCl_2_ was introduced to partially substitute PbBr_2_. For MnCl_2_ (manganese chloride, 99%, AR) doping, the ratio of PbBr_2_ and MnCl_2_ was set as 3:1, 3:2 and 1:1, and other experimental conditions were the same as the preparation of CsPbBr_3_ film. All operations are carried out in the atmosphere. In addition, CsBr and DMSO were provided by Shanghai Titan Scientific Co., Ltd. (Shanghai, China). PbBr_2_, EAC, OLA and MnCl_2_ were purchased from Shanghai Aladdin Biochemical Technology Co., Ltd. (Shanghai, China).

### 2.2. Characterizations for the As-Obtained Samples

The film’s surface morphology was measured by scanning electron microscopy (SEM, Quanta 200, FEI, Eindhoven, The Netherlands). The crystalline structure was characterized by X-ray diffraction (XRD, Ultima IV, Rigaku, Tokyo, Japan). An Ultraviolet-Visible-Infrared spectrophotometer (UV-vis-IR, UH 4150, Hitachi, Tokyo, Japan) was used to record the absorption spectra of the prepared films. The PL properties were measured by fluorescence spectrophotometer (FS5, Edinburgh Instruments, Edinburgh, UK). Energy dispersive X-ray spectroscopy (EDS, Genesis Apollo X, Pittsburgh, PA, USA) and X-ray photoelectron spectroscopy (XPS, Axis Ultra DLD, Kratos, Manchester, UK) were employed for the study of chemical constitution.

## 3. Results and Discussion

**Figure 1:**
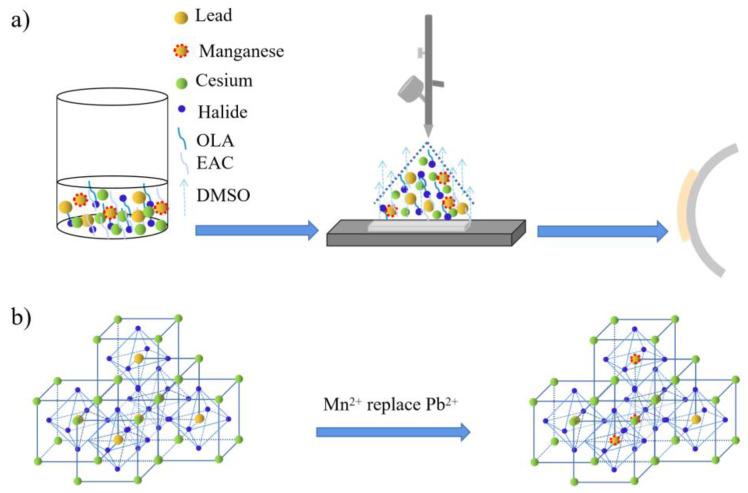
(**a**) The schematic diagram of solution spraying process; (**b**) Mn^2+^ replaced part of Pb^2+^.

In this work, the preparation diagram was described in Figure 1a. For CsPbBr_3_ fabrication, all reactants were sufficiently dissolved in DMSO solvent to prepare the precursor solution. In this solution, the mental ions Cs^+^, Pb^2+^ dispersed homogeneously and freely. Halide ions Br^−^ were released from mental halides of CsBr and PbBr_2_, then acted with OLA to form Br-OLA complexes. During the spraying process, the DMSO solvent was evaporated by the heating treatment. Br^−^ anions were separated from Br-OLA complexes and combined with Cs^+^, Pb^2+^ cations to form the expected CsPbBr_3_ products. Meanwhile, these CsPbBr_3_ products may be encapsulated and passivated by OLA molecules as suggested by previous works [30]. For CsPb_x_Mn_1−x_X_3_ film fabrication, Mn^2+^ replaced part of Pb^2+^ (as shown in Figure 1b) and Cl^−^ exchanged with part Br^−^ after MnCl_2_ adding in the precursor solution. Here, the partial substitution of Pb^2+^ by Mn^2+^ was driven by the halide exchange process of Br-to-Cl. During the Br-to-Cl exchange, the rigid octahedron structure of PbBr_6_^4−^ would be opened up, and then the Mn^2+^ would be incorporated into this structure to replace Pb^2+^. The above halide exchange driven cation exchange process has also been demonstrated by early studies [31].

**Figure 2:**
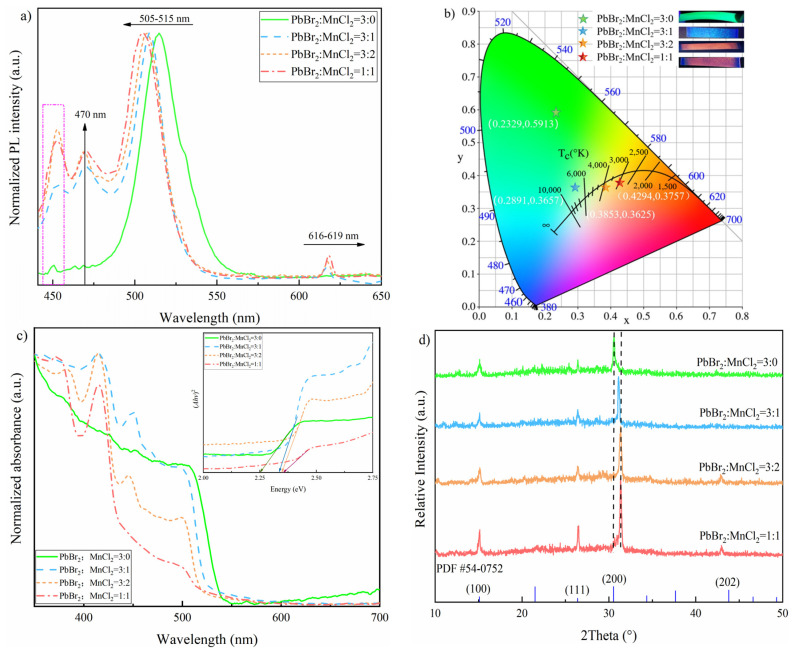
Optical properties of as-obtained samples prepared by different PbBr_2_/MnCl_2_ ratios: (**a**) PL spectra; (**b**) CIE diagram; (**c**) absorption spectra (inset plots of (*Ahv*)^2^ vs. (*hv*)); and (**d**) XRD pattern.

The PL spectra of CsPbBr_3_ and CsPb_x_Mn_1−x_(Br,Cl)_3_ films were shown in Figure 2a. For CsPbBr_3_ film, an emission band centered at 515 nm is observed, while the peaks at 450/470 nm could be attributed to EAC. When doped with MnCl_2_, two peaks at around 509/616 nm were observed. It was found that the main peak of 515 nm in pure CsPbBr_3_ film gradually blue-shifted to 505 nm with the ratio of PbBr_2_:MnCl_2_ increasing from 3:1 to 1:1. This may be caused by the band gap varying, which resulted from the increasing Br-to-Cl exchanging after MnCl_2_ incorporation [22,32]. The peak locating at 616 nm was derived from the energy transferring of ^4^T_1_→^6^A_1_ caused by Mn substitution for Pb in CsPb_x_Mn_1−x_(Br,Cl)_3_ material. In CsPb_x_Mn_1−x_(Br,Cl)_3_, after band to band optical excitation, the energy is transferred from the main material to the Mn^2+^ excited state [33]. In addition, the Mn^2+^ emission slightly red-shifted from 616 nm to 619 nm as the Mn ratio increased. This may be caused by the lattice contraction induced modification of Mn^2+^ ligand-field and the Mn^2+^ reabsorption/emission effect as suggested [22]. As observed, the PLQY values of the samples, prepared with different PbBr_2_: MnCl_2_ ratios of 3:0, 3:1, 3:2, 1:1, were 15.68%, 1.26%, 3.85% and 3.09%, respectively. It was found that the PLQY of MnCl_2_ doped samples displayed a downward trend when the PbBr_2_: MnCl_2_ ratio was over 3:2. This may be caused by a quenching effect due to excessive Mn. Figure 2b depicted the Commission Internationale de I’Eclairage (CIE) color space coordinates of CsPbBr_3_ and CsPb_x_Mn_1−x_(Br,Cl)_3_ films. For the CsPbBr_3_ sample, the CIE was (0.2329, 0.5913) and showed green light under 365 nm UV excitation. For the CsPb_x_Mn_1−x_(Br,Cl)_3_ sample, the corresponding CIE of different Pb/Mn ratios (3:1, 3:2, 1:1) were (0.2891, 0.3657), (0.3853, 0.3625), and (0.4294, 0.3757), respectively, and the as-prepared samples featured cyan, orange-red, pink-red separately. The cyan color for the sample fabricated under 3:1 Pb/Mn ratio may occur because the emission of main material was dominant while the energy transferring was in a low degree. With the increasing Mn content, the energy transferring was accelerated. This was well suggested by the color changing and the variation of normalized Mn^2+^ emission intensity. Additionally, the color purity was 53.8%, 20.1%, 24.4% and 41.6%, while the color temperature was 7344, 7839, 3749 and 2889 K, for 3:0, 3:1, 3:2 and 1:1 Pb/Mn ratio, respectively.

Furthermore, the absorbance spectra of as-obtained samples were determined by UV-vis spectrophotometer and displayed in Figure 2c. As observed, all samples had absorption edges around 510 nm, which was attributed to main materials CsPb(Br/Cl)_3_. Additionally, this edge bule-shifted gradually, with the increasing Cl^−^. The bandgap energy *Eg* of these samples were calculated by extrapolating the straight line of (*Ahv*)^2^ vs. (*hv*) plots displayed in the inset of Figure 2c. Here, A is absorbance, *h* is Planck’s constant, *v* is frequency. As observed, the *Eg* values for Mn-doped CsPbBr_3_ films (with the ratio of PbBr_2_:MnCl_2_ 3:0, 3:1, 3:2, 1:1) were 2.25, 2.33, 2.34 and 2.35 eV, separately. The crystal structure of prepared films was determined by XRD treatment as shown in Figure 2d. Regarding to the undoped sample, there were three peaks, locating around at 15.02°, 26.39°, and 30.56°, could be attributed to the (100), (111), and (200) planes of cubic CsPbBr_3_ (JCPDS 54-0752). This indicated the undoped sample was in pure perovskite phase. It was found that the diffraction peak of (202) plane gradually appeared at around 42.80° with MnCl_2_ doping increasing. Interestingly, the diffraction peak of (200) plane gradually shifted toward to higher angles as MnCl_2_ doping increased. As suggested by early studies [22], this was caused by the lattice contraction after the smaller Mn^2+^ (0.97 Å) and Cl^−^ incorporation into the lattice to separately replace Pb^2+^ (1.33 Å) and Br^−^. This is also confirmed by the changing of crystal size of prepared films. As calculated based on (200) plane by Scherrer formula, the crystal sizes corresponding to the different ratios of PbBr_2_:MnCl_2_ (3:0, 3:1, 3:2, 1:1) were 22.03, 21.31, 20.86, and 19.96 nm, respectively, which presented a downward trend.

**Figure 3:**
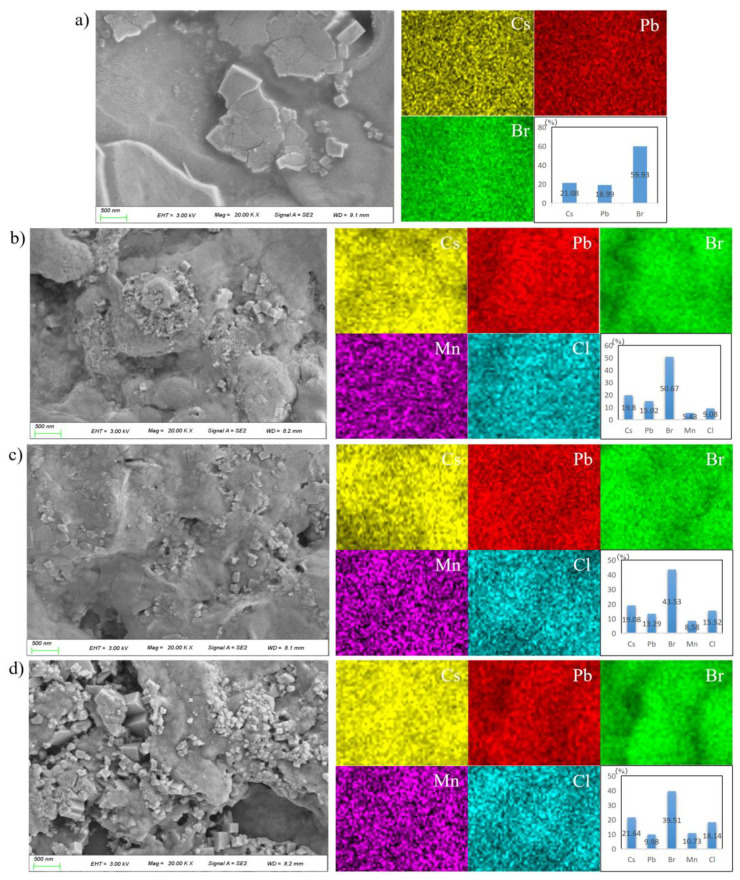
Morphologies and EDS mappings of the samples prepared by different PbBr_2_/MnCl_2_ ratios: (**a**) 3:0; (**b**) 3:1; (**c**) 3:2; (**d**) 1:1.

The surface morphologies of the samples prepared in 3:0, 3:1, 3:2 and 1:1 PbBr_2_:MnCl_2_ ratios were shown in Figure 3a–d, respectively. It was found that the undoped sample was in bulk-shape, and was dense and compact. This was benefitted from the Br-rich state as suggested by previous studies [34]. A few particles also appeared on the surface. As suggested by previous studies [30], the bulk-shaped or particle-shaped products were composed of agglomerated NCs by soft agglomeration of electrostatic force or Van der Waals force, especially in the presence of OLA. Interestingly, the dense and compact state of as-fabricated samples deteriorated as the MnCl_2_ ratio increased. More and more particles appeared as well as the pins. This was because Br was partially replaced by Cl to change the Br-rich state after MnCl_2_ doping. Thus, the morphology featured a deterioration trend with more Cl incorporating into the prepared film samples. Furthermore, the EDS mappings were also described in Figure 3a–d to determine the chemical composition of as-obtained samples prepared with different PbBr_2_:MnCl_2_ ratios. As observed, all elements including Cs, Pb/Mn, Br/Cl dispersed homogeneously on the sample surfaces. Concerning the Mn atom percent in prepared samples, the value increased from 0 to 10.73% with the Pb/Mn ratio varying from 3:0 to 1:1. The actual Pb/Mn ratios were 3:0, 2.8:1, 3.1:2 and 0.93:1, which were close to the expected values of 3:0, 3:1, 3:2 and 1:1. This indicated Pb was successfully substituted by Mn in host material as expected. In addition, the Cs:(Pb+Mn):(Cl+Br) ratio of fabricated samples was close to the stoichiometric value 1:1:3.

**Figure 4:**
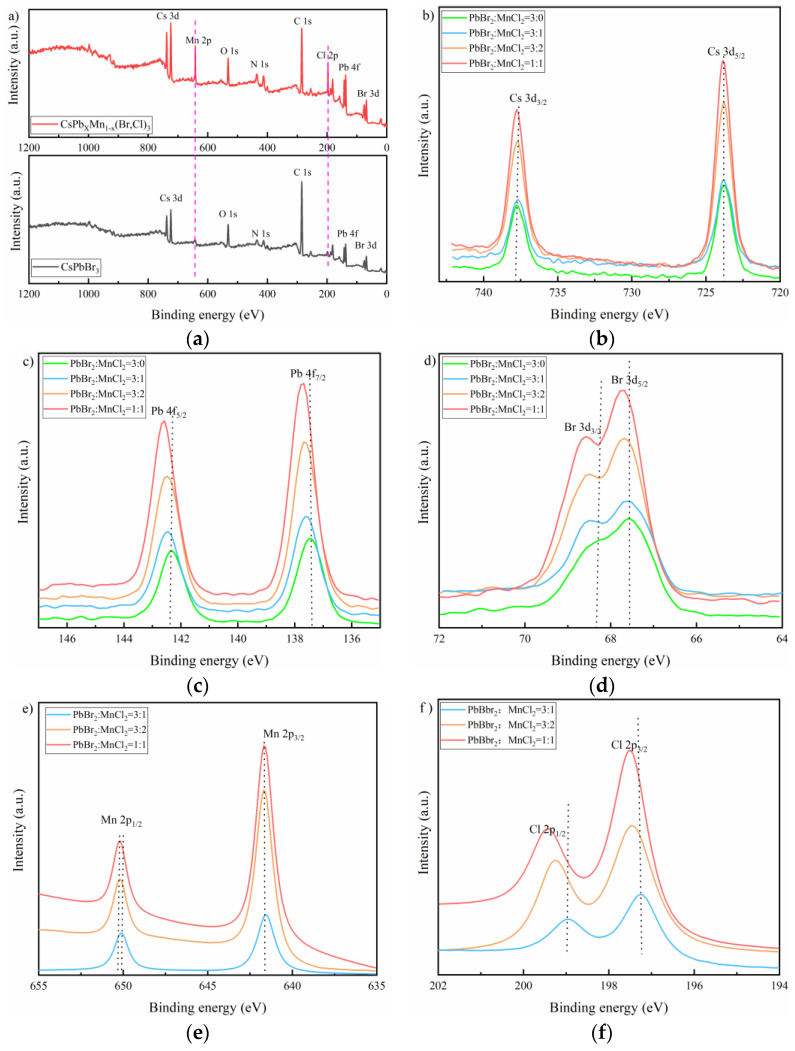
(**a**) XPS survey spectra of Mn-doped and undoped samples; the combining energy of different elements: (**b**) Cs 3d, (**c**) Pb 4f, (**d**) Br 3d, (**e**) Mn 2p, and (**f**) Cl 2p.

XPS survey was employed to further investigate the composition of as-prepared samples. As observed in Figure 4a, the orbital peaks of Mn 2p and Cl 2p appeared as expected in MnCl_2_ dopped sample comparing to the pure CsPbBr_3_ film. Cs, Pb and Br all had two split peaks locating at around 737.78 and 723.81 eV, 142.34 and 137.45 eV, and 68.45 and 67.56 eV, which were assigned to Cs 3d_3/2_ and Cs 3d_5/2_, Pb 4f_5/2_ and Pb 4f_7/2_, and Br 3d_3/2_ and Br 3d_5/2_ electronic levels, respectively, as displayed in Figure 4b–d [35]. Note that these peaks all slightly shifted to high binding energy as MnCl_2_ increased, especially for Pb 4f. This may be caused by the coordination bonding of Cs-Cl, Pb-Cl and Mn-Br on the surface of host material. This was favorable for the stability improvement of Cs 3d, Pb 4f and Br 3d. In addition, the obvious red-shifting of Pb 4f may also be a result from the changing of chemical environment and electron density after Mn doping as suggested by previous work [32,36]. Figure 4e showed two characteristic peaks located at 641.63 eV and 650.31 eV corresponding to Mn 2p_3/2_ and Mn 2p_1/2_, respectively. This indicated Mn was in divalent state, and also suggested Pb was successfully substituted by Mn as expected [35]. Similar to Cs and Pb, the binding energy of Mn 2p_1/2_ also slightly varied to a higher value as MnCl_2_ doping increased. For Cl, there were two peaks that appeared at 197.25 and 198.95 eV, as observed in Figure 4f.

**Figure 5:**
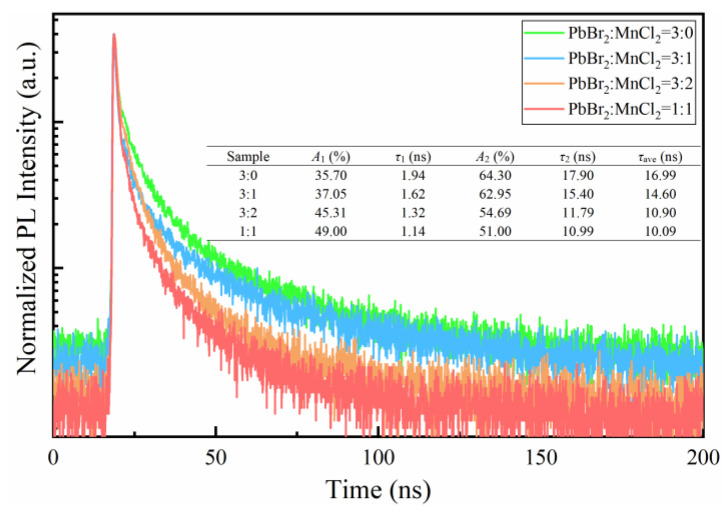
Time-resolved PL decay curves of the samples prepared by different PbBr_2_/MnCl_2_ ratios.

Figure 5 displayed the normalized time-resolved PL decay curves of as-prepared films. The curves were fitted by a bi-exponential function as shown in Equation (1):

(1) $$y=A_{0}+A_{1}\exp\left(-\frac{t}{\tau_{1}}\right)+A_{2}\exp\left(-\frac{t}{\tau_{2}}\right)\;$$

(2) $$\tau_{\mathrm{ave}}=\left(A_{1}\tau_{1}^{2}+A_{2}\tau_{2}^{2}\right)/\left(A_{1}\tau_{1}+A_{2}\tau_{2}\right)$$

where, *τ*_1_, *τ*_2_ are short-lived and long-lived lifetime originating from exciton radiative recombination and the trap-assisted nonradiative recombination, respectively. *A*_1_ and *A*_2_ are the corresponding occupied percent of *τ*_1_ and *τ*_2_, respectively. *A*_0_ is a constant, the average lifetime *τ*_ave_ is calculated by Equation (2). As observed, the values of *τ*_1_, *τ*_2_ and *τ*_ave_ all featured a downward trend as MnCl_2_ doping increased. It is worth mentioning that the value of *τ*_1_ decreased from 1.94 ns to 1.14 ns, which may be derived from the bandgap increasing caused by Br-to-Cl anion exchange [22]. Moreover, the occupied percent of *τ*_1_ was increased from 35.70% to 49.00% which meant a higher ratio of radiative to nonradiative recombination. Above indicated a better exciton emission and less defect-related transition, as suggested by previous studies [30].

## 4. Conclusions

In conclusion, a CsPb_x_Mn_1−x_(Br,Cl)_3_ film was successfully prepared by facile solution spraying approach with flexible PET substrate through MnCl_2_ doping in host CsPbBr_3_ material. As the results showed, the green emission in CsPbBr_3_ film gradually transferred to be cyan, orange-red, and pink-red as MnCl_2_ increased, which was derived from the energy transferring between host material and Mn^2+^ excited state. It was found that the dense and compact film was varied to be a coexisting state of bulk and nanoparticles products with the MnCl_2_ increasing in doped material. The EDS mapping displayed that all chemical dispersed uniformly on the film surface, and Pb was successfully substituted by Mn as expected, which was further confirmed by XPS investigation. Moreover, with MnCl_2_ doping rising, the combining energy of Cs, Pb, and Mn featured a red-shifting variation which may be caused by the formation of Cs-Cl, Pb-Cl and Mn-Br coordination bonding. Time-resolved PL decay study indicated the weight of short-lifetime presented an upward trend as doping increased, which suggested a better exciton emission and less defect-related transition.

## Data Availability

Not applicable.
